# Radical carbonylations using a continuous microflow system

**DOI:** 10.3762/bjoc.5.34

**Published:** 2009-07-13

**Authors:** Takahide Fukuyama, Md Taifur Rahman, Naoya Kamata, Ilhyong Ryu

**Affiliations:** 1 Department of Chemistry, Graduate School of Science, Osaka Prefecture University, Sakai, Osaka 599-8531, Japan

**Keywords:** continuous flow system, microreactor, radical carbonylation, radical mediator, V-65

## Abstract

Radical-based carbonylation reactions of alkyl halides were conducted in a microflow reactor under pressurized carbon monoxide gas. Good to excellent yields of carbonylated products were obtained via radical formylation, carbonylative cyclization and three-component coupling reactions, using tributyltin hydride or TTMSS as a radical mediator.

## Introduction

Placing a reaction mixture fluid inside a microstructured channel network helps gain a high surface area to volume ratio that in turn ensures rapid heat and mass transfer [1–3]. Precise control of reaction temperature and residence time, excellent mixing properties for reactants/reagents as well as a flow nature of the microreaction system, often result in higher conversion, greater yields, superior product selectivity and safety. In recent years, the potential of this new technology for organic synthesis have been recognized and many applications have been demonstrated by our group [4–6] as well as others [7–11].

**Scheme 1:**
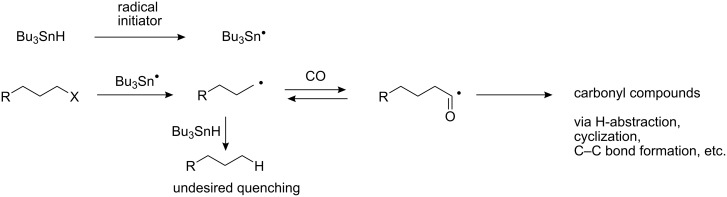
Tin-mediated radical carbonylation and the competing reduction.

In our recent report, we demonstrated that excellent thermal efficiency of the microflow system would lead to effective execution of tin hydride and TTMSS (tris(trimethylsilyl)silane)-mediated radical reduction and cyclization reactions [12]. The Seeberger group reported reduction and hydrosilylation using TTMSS in a microflow system [13]. In our study, we found that the combination of a rapidly decomposing radical initiator with a microreaction device allowed the reactions to be completed in a very short period of time giving good yields of the desired products. Carbon monoxide is an important feedstock and our group has actively pursued carbonylation reactions [14–17]. Thermally induced radical carbonylations using tin hydride usually require pressurized CO conditions to ensure that the concentration of CO around the radical centers is high enough to compete with the premature quenching by tin hydride (Scheme 1). Encouraged by our previous successes with both radical reactions [12] and metal-catalyzed carbonylation reactions using microflow devices [18–20], we decided to examine a continuous microreaction system for radical carbonylation reactions, for which we typically used stainless-steel autoclaves as the reactor in batch systems.

## Results and Discussion

**Figure 1:**
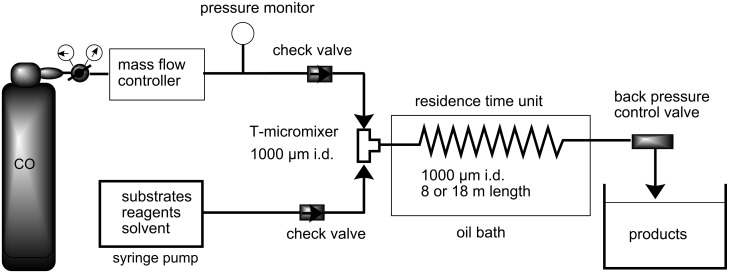
Microflow system available for radical carbonylation using pressurized CO.

The microflow system we employed was simple, yet robust enough to withstand high CO pressures (ca. 80 atm) (Figure 1). A metered stream of CO gas was fed in a controlled manner into the system and was mixed with the toluene solution containing a radical mediator, a radical initiator and a substrate in a T-shaped micromixer (stainless steel, internal diameter: 1000 μm). This biphasic (gas-liquid) mixture was then guided through a stainless steel tubular reactor (internal diameter: 1000 μm), acting as the residence time unit (RTU), under heated conditions using an oil bath. A back pressure control valve was connected at the end of the RTU to regulate and maintain the pressure of the reactor system. Reaction time was adjusted via the flow rates of CO and the liquid.

Using the microflow system depicted by Figure 1, we carried out the radical formylation of 1-bromododecane (**1**) with CO in the presence of tributyltin hydride (bath temp. 80 °C) [21]. However, we encountered incomplete conversion of the starting bromide when we used AIBN (2,2′-azobisisobutyronitrile) as a radical initiator. This was attributed to the rather slow decomposition rate of the AIBN (half-life time: 90 min at 85 °C) in light of the time frame employed for the microflow reaction (residence time, 12 min). When we switched AIBN to a more rapidly decomposing V-65 (2,2′-azobis(2,4-dimethylvaleronitrile), half-life time: 12 min at 80 °C), 100% conversion was attained to give tridecanal (**2**) in a 77% yield (Table 1, entry 1). Microflow carbonylation of bromocyclohexane (**3**) and 1-bromoadamantane (**5**) also worked well to furnish the corresponding aldehydes **4** and **6** in good yields (entries 2 and 3). The judicious choice of a radical initiator is important for high conversion in a short reaction time for carbonylation reactions, which is in accordance with our previous experience with microflow radical reduction and cyclization of organic halides using tributyltin hydride [12].

**Table 1:** Radical carbonylation in a microflow system.^a^

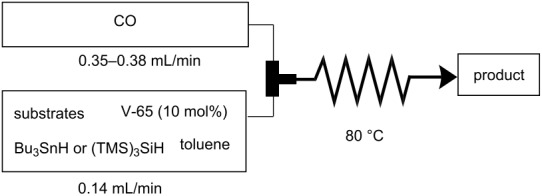			
entry	substrate	conditions	product/yield^b^

1	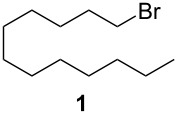	0.02 M in toluene, Bu_3_SnH (1.2 equiv), V-65 (10 mol%), CO 83 atm 12 min	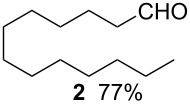
2	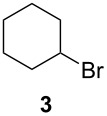	0.02 M in toluene, Bu_3_SnH (1.2 equiv), V-65 (10 mol%), CO 85 atm 12 min	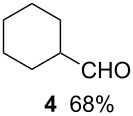
3	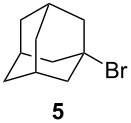	0.02 M in toluene, Bu_3_SnH (1.2 equiv), V-65 (10 mol%), CO 85 atm 12 min	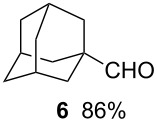
4	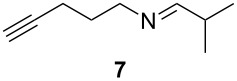	0.05 M in toluene, Bu_3_SnH (1.1 equiv), V-65 (10 mol%), CO 79 atm 27 min	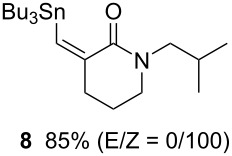
5	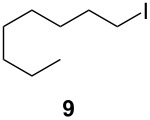	0.017 M in toluene, Bu_3_SnH (1.5 equiv), H_2_C=CH-COCH_3_ (4 equiv) V-65 (10 mol%), CO 85 atm 29 min	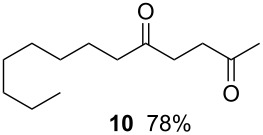
6	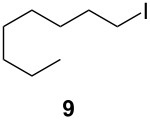	0.025 M in toluene, (TMS)_3_SiH (1.5 equiv), H_2_C=CH-COCH_3_ (1.2 equiv) V-65 (30 mol%), CO 20 atm 30 min	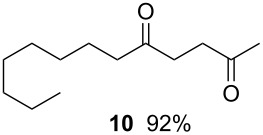
7	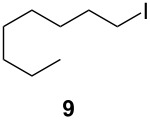	0.025 M in toluene, (TMS)_3_SiH (1.5 equiv), H_2_C=CH-CN (1.2 equiv) V-65 (30 mol%), CO 20 atm 30 min	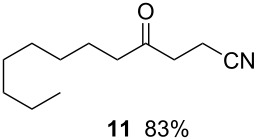

^a^Conditions: substrate (1 mmol, 0.017–0.05 M in toluene), Bu_3_SnH or TTMSS (1.1–1.5 equiv), V-65 (10–30 mol%), CO (20–85 atm), T-shaped micromixer (1000 μm i.d.), residence time unit (1000 μm i.d. length: 8 m for entries 1–3, 18 m for entries 4–7). ^b^Yields were determined by GC analysis using decane as an internal standard.

Tin-mediated radical carbonylation has a wide range of applications including carbonylative cyclization and multi-component coupling reactions. In this regard, we again chose stannylcarbonylation using 1,6-azaenyne **7** as a model for cyclization [22–23]. Using a similar microflow system but with an extended reaction time, we were able to obtain the desired six-membered ring lactam **8** in good yield (entry 4). Using a similar microflow system, we then carried out a three-component coupling reaction comprised of 1-iodooctane (**9**), CO, and methyl vinyl ketone in the presence of tributyltin hydride [24], which also worked well to give 2,5-tridecandione (**10**) in 78% yield (entry 5).

Since tris(trimethylsilyl)silane (TTMSS) delivers a hydrogen atom to a carbon-centered radical at a slower rate than tributyltin hydride [25], carbonylation reactions with TTMSS can be carried out at lower CO pressure without being plagued by the premature reduction of the key radical. Hence, we anticipated that the combination of low pressure CO/V-65 could be successfully applied to the TTMSS-mediated three component carbonylative-coupling reaction of 1-iodooctane (**9**) [26–27] in a microflow system using methyl vinyl ketone and acrylonitrile as acyl radical traps (entries 6 and 7 ). Gratifyingly, in both cases, good yields of the three-component coupling products were formed by using reduced CO pressure.

## Conclusion

We have developed a facile platform to conduct radical carbonylation under CO pressure in a flow system comprised of a T-shaped mixer and a tabular residence time unit. Using V-65 as a radical initiator, we were able to carry out typical tin- or silicon-based radical carbonylation reactions leading to aldehydes, unsymmetrical ketones, and a lactam, in a continuous microflow system.

## Experimental

**Typical procedure for radical carbonylation in a microflow system. The radical formylation of 1-bromododecane (1).** 1-Bromododecane (**1**, 1 mmol, 249.5 mg), V-65 (0.1 mmol, 24.8 mg), Bu_3_SnH (1.2 mmol, 352.9 mg), and decane (59 mg) as an internal standard were dissolved in toluene (50 mL). The toluene solution was placed in a syringe (17 mL), which was then attached to a syringe pump. The system was pressurized with CO (83 atm) by means of the pressure control valve. The flow rate of CO was controlled at 0.14 mL/min by the mass flow controller. The toluene solution was introduced at a flow rate of 0.37 mL/min, then was mixed with CO in the T-shaped micromixer (i.d. = 1000 μm). The reaction was then fed into the residence time unit, which was immersed in an oil bath and heated at 80 °C. The time needed for the reaction mixture to travel through the residence time unit was expressed as the residence time (12 min). The mixture of products was collected at the outlet. The initial effluent exiting the microflow reactor was discarded (ca. 20 min) until a stable gas-liquid mixing was achieved, and the following portion was collected for a 10 min period. The yield was determined by GC analysis, and the product was identified by comparison of ^1^H NMR spectrum and a retention time for GC analysis with those of authentic sample.
